# Treatment of Nodular Lymphocyte-Predominant Hodgkin Lymphoma: Where Do We Stand? Where Do We Go?

**DOI:** 10.3390/cancers15133310

**Published:** 2023-06-23

**Authors:** Dennis A. Eichenauer, Michael Fuchs

**Affiliations:** 1 First Department of Internal Medicine, Center for Integrated Oncology Aachen Bonn Cologne Dusseldorf, University of Cologne, D-50937 Cologne, Germany; michael.fuchs@uk-koeln.de; 2 German Hodgkin Study Group (GHSG), First Department of Internal Medicine, University Hospital Cologne, D-50937 Cologne, Germany

**Keywords:** nodular lymphocyte-predominant Hodgkin lymphoma, B-NHL, radiotherapy, chemotherapy, anti-CD20 antibody treatment, histopathological growth pattern

## Abstract

**Simple Summary:**

Nodular lymphocyte-predominant Hodgkin lymphoma (NLPHL) is a rare lymphoma entity accounting for ≈5% of all Hodgkin lymphoma (HL) cases. Since pathological characteristics and clinical presentation of NLPHL differ from classical HL and often resemble indolent B-cell non-Hodgkin lymphoma (B-NHL), nodular lymphocyte-predominant B-cell lymphoma has been proposed as an alternative name. In recent years, NLPHL has increasingly been treated with B-NHL-directed approaches, although more data are available on HL-directed treatment strategies. Overall, the outcomes of NLPHL patients are excellent, and the excess mortality in comparison with the general population is low. Future research in NLPHL will thus aim to determine strategies to reduce treatment intensity whenever possible. The definition of the optimal use of HL-directed and B-NHL-directed approaches in this disease is also pending.

**Abstract:**

Nodular lymphocyte-predominant Hodgkin lymphoma (NLPHL) is a rare B cell-derived lymphoma entity accounting for ≈5% of all Hodgkin lymphoma (HL) cases. In recent decades, patients with newly diagnosed NLPHL have usually been treated very similarly to classical HL (cHL). The 10-year overall survival rates with HL-directed approaches are in excess of 90%. However, pathological and clinical characteristics of NLPHL resemble indolent B-cell non-Hodgkin lymphoma (B-NHL) in some aspects. Thus, nodular lymphocyte-predominant B-cell lymphoma has been proposed as an alternative name, and the use of B-NHL-directed treatment strategies has become more common in NLPHL despite limited data. Given the often indolent clinical course of NLPHL, even in the case of relapse, the majority of patients with disease recurrence do not require high-dose chemotherapy and autologous stem cell transplantation but are treated sufficiently with low-intensity approaches such as single-agent anti-CD20 antibody treatment. The establishment of novel prognostic scores for NLPHL patients may optimize risk group and treatment allocation in newly diagnosed and relapsed disease.

## 1. Introduction

Nodular lymphocyte-predominant Hodgkin lymphoma (NLPHL) is a rare B-cell lymphoma with an incidence of approximately 0.1–0.2/100.000/year. Roughly 75% of patients are male, and the median age at initial diagnosis is around 40 years [1]. Pathological and clinical characteristics of NLPHL differ from classical Hodgkin lymphoma (cHL) in some aspects. 

The malignant lymphocyte predominant (LP) cells in NLPHL are, in contrast to the disease-defining Hodgkin and Reed–Sternberg (H-RS) cells in cHL, consistently positive for CD20 but lack CD30 and CD15 [1,2]. The frequency of positivity for Epstein–Barr virus (EBV) represents another difference between LP cells and H-RS cells. While H-RS cells are positive for EBV in a relevant proportion of patients, EBV can be detected in LP cells in less than 5% of cases [3,4]. In terms of histopathology, six distinct histopathological growth patterns were described by Fan and colleagues. Typical growth patterns (patterns A and B according to Fan et al.; ≈75% of cases) can be distinguished from atypical growth patterns (patterns C–F according to Fan et al.; ≈25% of cases) [5,6]. Atypical growth patterns are associated with more advanced disease, a higher recurrence rate, and a shorter median time to relapse [6,7,8]. Cases presenting with growth pattern E are sometimes difficult to differentiate from T cell and histiocyte-rich large B-cell lymphoma [9]. The microenvironment of cases presenting with growth pattern E also contains more CD163-positive macrophages than cases presenting with other atypical and typical growth patterns, respectively [7]. 

The clinical course of NLPHL is usually indolent, and patients present with early stage disease in most cases [10]. The long-term prognosis of NLPHL patients is excellent, and the excess mortality in comparison with the general population is low [11]. However, approximately 10% of patients develop histological transformation into aggressive B-NHL within 10 years from the initial NLPHL diagnosis [12,13]. Given these similarities with indolent B-NHL, the Clinical Advisory Committee has proposed nodular lymphocyte-predominant B-cell lymphoma as an alternative name [14]. In contrast, the most recent version of the WHO classification has maintained the name NLPHL [15]. 

The present review article aims at summarizing the available data on current treatment strategies and possible future approaches in NLPHL.

## 2. First-Line Treatment

The risk group allocation in NLPHL is very similar to cHL. Hence, patients are divided into three groups (early stage favorable, early stage unfavorable, and advanced stage). However, the early stage favorable group is further subdivided, and patients with stage IA disease without clinical risk factors usually receive less intensive treatment. 

### 2.1. Treatment of Stage IA NLPHL without Clinical Risk Factors

Adults with stage IA NLPHL without clinical risk factors are mostly treated with radiotherapy (RT) alone. Different studies have indicated outstanding progression-free survival (PFS) and overall survival (OS) rates with this approach which could not be improved by the addition of chemotherapy. The largest analysis on the outcomes of patients with stage IA NLPHL without clinical risk factors was conducted by the German Hodgkin Study Group (GHSG). Patients had 8-year PFS rates of 88.5%, 84.3%, and 91.9% and 8-year OS rates of 98.6%, 95.7%, and 99.0% after combined-modality treatment (CMT) (*n* = 72), extended-field radiotherapy (EF-RT) alone (*n* = 49), and involved-field radiotherapy (IF-RT) alone (*n* = 108), respectively [16]. Grade III/IV acute toxicities such as leukopenia, infection, and hair loss occurred more frequently after CMT than after EF-RT and IF-RT [17]. Similar results were obtained from a retrospective study conducted at a large single institution in the United States. Again, disease control and OS were excellent irrespective of whether extended RT, regional RT, or limited RT had been applied (Table 1) [18]. Given these results, different guidelines have adopted limited-field RT alone as standard treatment for adult patients with stage IA NLPHL without clinical risk factors [19,20]. In children and adolescents, stage IA NLPHL with only one affected lymph node is often treated with resection alone. This treatment strategy has been investigated in the prospective AHOD03P1 study conducted by the Children’s Oncology Group. In this study, pediatric patients diagnosed with NLPHL affecting a single lymph node did not receive consolidation chemotherapy or RT if they had complete metabolic remission after lymph node surgery according to computed tomography (CT) and positron emission tomography (PET). A total of 52 patients were eligible for this approach. The 5-year event-free survival was 77.1%. There were no deaths during the observation period [21]. Based on these data, several guidelines recommend resection alone for children with stage IA NLPHL in case they have achieved complete metabolic remission after lymph node surgery [22,23].

### 2.2. HL-Directed First-Line Treatment of NLPHL

Historically, first-line treatment of NLPHL has been very similar to cHL, and patients with NLPHL have been treated in the same studies as patients with cHL. Individuals with early stage favorable disease, who represent the majority of NLPHL cases, mostly receive CMT consisting of brief chemotherapy followed by consolidation RT. An older retrospective analysis using the British Columbia Cancer Agency (BCCA) database indicated 10-year PFS and OS rates of 91% and 93%, respectively, with this approach. The most common chemotherapy protocol applied in patients included in this analysis was ABVD (doxorubicin, bleomycin, vinblastine, and dacarbazine) [25]. Another retrospective analysis conducted by the GHSG included 251 patients with newly diagnosed early stage favorable NLPHL who had treatment within the randomized HD7, HD10, and HD13 studies. The majority of patients received two cycles of ABVD followed by consolidation RT. The 10-year PFS and OS rates were 79.7% and 93.3%, respectively (Table 1) [26]. The more recent randomized HD16 study for early stage favorable HL included 85 NLPHL patients. All patients received two cycles of ABVD chemotherapy before an interim PET was performed. Patients with a positive interim PET received consolidation RT whereas patients with a negative PET either had consolidation RT or no further treatment depending on whether they were assigned to the standard arm or the experimental arm of the study. At 5 years, the PFS and OS rates for all 85 patients were 90.3% and 100%, respectively. Patients with a negative interim PET who did not have consolidation RT tended strongly towards an impaired PFS in comparison with interim PET-negative patients receiving standard CMT [32].

Only a small proportion of NLPHL patients present with early stage unfavorable disease. Data on this patient group are thus limited. However, an analysis comprising 76 individuals with early stage unfavorable NLPHL treated within the randomized GHSG HD8, HD11, and HD14 studies revealed excellent outcomes with HL-directed approaches consisting of chemotherapy with ABVD or different BEACOPP (bleomycin, etoposide, doxorubicin, cyclophosphamide, vincristine, procarbazine, and prednisone) variants plus consolidation RT. The 10-year PFS and OS rates were 72.1% and 96.2%, respectively (Table 1) [26]. 

Standard approaches in the treatment of advanced cHL have also been applied in individuals with advanced NLPHL. However, the most common chemotherapy protocols in advanced cHL, i.e., ABVD and escalated BEACOPP, may not be optimal for the treatment of advanced NLPHL for different reasons. NLPHL patients treated with ABVD have a higher lymphoma recurrence rate than their counterparts with cHL. According to a matched-pair analysis including 42 patients with advanced NLPHL and 84 patients with advanced cHL mostly treated with ABVD or ABVD-like protocols, the time to progression, defined as the time from the date of diagnosis to the date of relapse or death from any lymphoma, was significantly impaired for patients with NLPHL due to an increased rate of lymphoma recurrence with either NLPHL histology or histological transformation into aggressive B-NHL [28]. In contrast to ABVD, treatment with escalated BEACOPP appears to result in better disease control. A total of 144 patients with advanced NLPHL were treated within the randomized GHSG HD9, HD12, and HD15 studies and mostly received BEACOPP-based chemotherapy optionally followed by localized RT. At 10 years, PFS and OS rates were 69.8% and 87.4%, respectively (Table 1) [26]. The more recent randomized HD18 trial for advanced HL evaluated treatment guidance based on the result of an interim PET after two cycles of escalated BEACOPP. A total of 84 NLPHL patients were included in this study. Of these, the majority had a negative interim PET and were randomized between standard treatment with a total of at least six cycles of escalated BEACOPP and reduced treatment with a total of only four cycles. The treatment reduction did not result in a loss of disease control, so patients with advanced NLPHL and a negative interim PET after two cycles of escalated BEACOPP were sufficiently treated with a total of 4 cycles of chemotherapy. Patients with a positive interim PET after two cycles of escalated BEACOPP were randomized between either standard treatment alone or standard treatment plus rituximab. Although final conclusions are difficult to draw due to the limited number of NLPHL patients treated with this approach, the addition of rituximab to escalated BEACOPP did not seem to result in a significant improvement in disease control in this patient group. The 84 NLPHL patients treated within the HD18 study had 5-year PFS and OS rates of 82.4% and 94.8%, respectively [33]. Despite these encouraging PFS and OS rates, only a minority of patients with advanced NLPHL are candidates for intensive treatment with escalated BEACOPP given the more indolent clinical course, the lower lymphoma burden, and the smaller proportion of patients that die from lymphoma-related causes in comparison with cHL on the one hand and the increased rate of toxicities associated with this intensive protocol on the other hand [26,33]. 

### 2.3. Rituximab-Containing and NHL-Directed First-Line Treatment of NLPHL

Data on outcomes of NLPHL patients treated with rituximab either alone or in combination with conventional chemotherapy have become available in recent years.

A phase II study conducted by the GHSG aimed at minimizing the risk for the development of toxicities in patients with stage IA NLPHL without clinical risk factors. Thus, treatment was limited to four weekly standard doses of rituximab. All 28 patients included in the study responded to treatment. No grade III/IV toxicities were reported [34]. However, the long-term disease control was inferior to RT alone, with a 10-year PFS of only 51.1%. The 10-year OS was 91.0% [29]. Another phase II study investigating rituximab administered as single agent was conducted in the United States. Patients with newly diagnosed and relapsed NLPHL were eligible. Treatment consisted of either four weekly standard doses of rituximab alone or four weekly standard doses followed by rituximab maintenance therapy every 6 months for 2 years. The response rate among the 21 patients with newly diagnosed disease included in the study was 100%. The 5-year PFS rates were 41.7% for patients treated with rituximab alone and 51.9% for patients receiving rituximab induction followed by rituximab maintenance. The 5-year OS was 100% for both treatment groups (Table 1) [30]. 

The combination of rituximab and conventional chemotherapy has been investigated in some retrospective studies. An analysis from Italy included 16 NLPHL patients who received rituximab-containing treatment, i.e., rituximab alone or rituximab in combination with ABVD. The outcomes of these patients were compared to a historical control consisting of 12 individuals who received rituximab-free treatment consisting of chemotherapy with ABVD optionally followed by consolidation RT. At 7 years, patients receiving rituximab-containing treatment had a superior PFS. However, subgroup analysis indicated a PFS advantage only for patients with advanced-stage disease, whereas rituximab-containing treatment did not result in a PFS advantage for patients with early stage favorable and early stage unfavorable disease. The 7-year OS did also not differ between the groups receiving rituximab-containing and rituximab-free treatment, respectively [35]. A more recent large multi-institutional retrospective analysis conducted by the Fondazione Italiana Linfomi also aimed at evaluating the role of rituximab in the first-line treatment of NLPHL. A total of 308 patients treated at 20 centers in Italy were included. Of these, 193 had stage II-IV disease and received either chemotherapy alone (*n* = 81) or chemotherapy plus rituximab (*n* = 112). Systemic treatment in the chemotherapy alone group consisted of ABVD in all but one patient, whereas patients in the chemotherapy plus rituximab group had either ABVD (*n* = 66) or CHOP (cyclophosphamide, doxorubicin, vincristine, and prednisone) (*n* = 46). The 5-year PFS rate for the patients receiving rituximab-containing treatment was 89.6% and thus significantly better than for the patient group treated without rituximab (5-year PFS rate: 72.7%). Among the patients who had chemotherapy plus rituximab, no PFS difference was detected between those who had ABVD and those who had CHOP. The 5-year OS rates for patients receiving rituximab-containing and rituximab-free approaches were 98.8% and 95.0%, respectively, and did thus not differ between the groups (Table 1) [27].

Outcomes of NLPHL patients receiving BR (bendamustine and rituximab) have been reported initially in a small case series comprising nine patients. Of these, seven patients had newly diagnosed disease. All patients responded to treatment. After a median observation time of 34 months, no case of relapse occurred [36]. A follow-up analysis with a median observation time of 74 months included 20 patients, of whom 15 received BR as first-line treatment. Again, there were no cases of disease recurrence. A total of two patients died due to causes not related to NLPHL (Table 1) [31]. Although data on the use of the R-CVP (rituximab, cyclophosphamide, vinblastine, and prednisone) protocol in NLPHL are limited, this regimen is frequently used as first-line treatment, as revealed by an international survey conducted by the Global NLPHL One Working Group [37]. This is likely due to the good tolerability and the promising results obtained with CVP in children and adolescents with NLPHL [38]. 

An analysis using the Swedish Lymphoma Registry included 158 patients who had been diagnosed with NLPHL between 2000 and 2014. It investigated trends in the use of rituximab over time. The proportion of patients receiving rituximab either alone or in combination with systemic treatment increased from 3% between 2000 and 2004 to 54% between 2010 and 2014. The increase was more pronounced in patients with stage IIB-IV disease (increase from 7% between 2000 and 2004 to 100% between 2010 and 2014) than in patients with stage I-IIA disease (increase from 2% between 2000 and 2004 to 30% between 2010 and 2014). The more frequent use of rituximab correlated with a significant improvement in terms of OS in the most recent period [39]. However, it has not been reported whether the OS improvement held true irrespective of the stage at NLPHL diagnosis or whether it was restricted to certain subgroups. 

## 3. Treatment of Relapsed NLPHL and Histological Transformation

Patients with NLPHL experience disease recurrence slightly more often than patients with cHL. In particular, late relapses are more common. While the prognosis of patients with late relapse is still excellent, patients with disease recurrence within 24 months from the initial NLPHL diagnosis have an impaired OS. According to an analysis from the GHSG, including 471 NLPHL patients who had stage-adapted first-line treatment within the randomized HD7-HD15 studies, patients who never developed disease recurrence or relapsed more than 24 months after the initial NLPHL diagnosis had 10-year OS rates of 93.6% and 95.9%, respectively. In contrast, the 10-year OS rate for patients with disease recurrence less than 24 months after the initial NLPHL diagnosis was only 47.1% [26]. The time to relapse thus has a significant prognostic impact and represents one of the factors that guide second-line treatment in NLPHL. Additional factors are the previous treatment as well as lymphoma burden and clinical presentation at disease recurrence.

### 3.1. Low-Intensity Treatment for Relapsed NLPHL

Patients with late NLPHL recurrence, low lymphoma burden, and no or little symptoms at relapse are often treated sufficiently with low-intensity approaches such as single-agent anti-CD20 antibody treatment. A small phase II study conducted by the GHSG included 15 patients with relapsed NLPHL. Treatment consisted of four weekly standard doses of rituximab. The overall response rate was 94%. After a median observation time of 63 months, the median time for disease progression was 33 months. One patient died during follow-up [40]. A phase II study conducted in the United States included 18 patients with NLPHL recurrence. Patients either received four weekly rituximab doses alone or four weekly rituximab doses followed by rituximab maintenance every 6 months for 2 years. All patients responded to treatment. The 5-year PFS rates were 36.4% for patients treated with four doses of rituximab alone and 71.4% for patients receiving rituximab induction followed by rituximab maintenance. The 5-year OS rates for the treatment groups were 90.9% and 71.4%, respectively [30]. Comparable results were obtained with the second-generation anti-CD20 antibody ofatumumab in a phase II study, including a total of 28 patients with relapsed NLPHL. Treatment consisted of eight weekly doses. The objective response rate was 96%. After a median follow-up of 26 months, the 1-year PFS rate was 93%, and the PFS at 2 years was 80%. No patient died during observation (Table 2) [41]. Single-agent treatment with the Bruton´s tyrosine kinase inhibitor ibrutinib was also investigated in a phase II study for individuals with relapsed NLPHL. A total of 16 patients were included and received ibrutinib at a daily dose of 560 mg for a maximum of 60 weeks. At a median follow-up of 25 months, the 18-month PFS rate was 56.3%. Thus, disease control with this approach was worse than with single-agent anti-CD20 antibody treatment [42].

### 3.2. Conventional-Dose and High-Dose Chemotherapy as Salvage Treatment in Relapsed NLPHL

In patients with NLPHL recurrence after RT alone and in those who only had limited amounts of chemotherapy during first-line treatment, stage-adapted conventional chemotherapy optionally combined with an anti-CD20 antibody and RT can be considered as salvage treatment. Among 99 patients with relapsed NLPHL included in a retrospective analysis conducted by the GHSG, 27 had second-line treatment comprising conventional chemotherapy. The 5-year PFS and OS estimates for these patients were 68.0% and 77.8%, respectively (Table 2) [43]. 

High-dose chemotherapy and autologous stem cell transplantation (ASCT) can be restricted to a minority of patients with relapsed NLPHL, i.e., those presenting with risk factors such as a short time interval between first-line treatment and disease recurrence. According to retrospective analyses on relapsed NLPHL from the GHSG and two large cancer centers in North America, respectively, only 31% and 20% of patients received high-dose chemotherapy followed by ASCT as second-line treatment. The 5-year PFS rates with this approach were 84.6% in the GHSG analysis and 83.0% in the analysis from North America [43,44]. The largest analysis on the use of high-dose chemotherapy and ASCT in relapsed NLPHL was performed by the European Society for Blood and Marrow Transplantation. Overall, 60 patients were taken into account. The median time between NLPHL diagnosis and ASCT was 21 months. Patients presented with stage III/IV disease in 63% of cases. After a median observation time of 56 months, the 5-year PFS and OS rates were 66% and 87%, respectively (Table 2) [45].

### 3.3. Treatment of Patients with Transformation into Aggressive B-NHL

Approximately 10% of NLPHL patients develop histological transformation into aggressive B-NHL within 10 years from the initial lymphoma diagnosis [12,13]. Splenic involvement at the initial NLPHL diagnosis appears to represent the major risk factor for histological transformation [13,47]. Once the diagnosis of histological transformation has been made, treatment is very similar to de novo aggressive B-NHL. Patients who received no or only limited amounts of chemotherapy as part of their treatment for NLPHL are candidates for R-CHOP. The International Lymphoma Radiation Oncology Group conducted a large multi-institutional retrospective analysis including 559 patients with stage I/II NLPHL. First-line treatment of these patients often consisted of RT alone or CMT with only a few chemotherapy cycles. During follow-up, 21 patients developed histological transformation into aggressive B-NHL. Treatment at histological transformation most frequently consisted of R-CHOP. The 5-year PFS and OS rates after histological transformation were 62.2% and 88.4%, respectively [24]. Among patients with more extensive prior treatment for NLPHL, high-dose chemotherapy and ASCT represent the most common approach to histological transformation. An analysis from the United Kingdom revealed that the long-term PFS and OS rates for patients treated with high-dose chemotherapy and ASCT for histological transformation into aggressive B-NHL were in excess of 60% [48].

## 4. Conclusions and Future Directions

At present, patients with early-stage NLPHL are treated with limited-field RT alone in case of stage IA disease without clinical risk factors and CMT consisting of a brief chemotherapy (two cycles of ABVD in early-stage favorable disease and four cycles in early-stage unfavorable disease) followed by limited-field consolidation RT in case of early-stage favorable and early-stage unfavorable disease, respectively. Data on the optimal use of B-NHL-directed approaches in early-stage NLPHL have not been available until now. In advanced NLPHL, first-line treatment with B-NHL-directed protocols such as R-CHOP appears more appropriate than the use of HL-directed protocols such as ABVD and escalated BEACOPP in many cases. This is due to the reduced disease control with ABVD (at least if administered without rituximab) and the often unfavorable risk–benefit ratio with escalated BEACOPP (a relevant proportion of patients may be overtreated with this intensive protocol as the extent of disease is commonly limited even in cases with advanced NLPHL). In relapsed NLPHL, treatment is chosen individually based on factors such as the time interval between the initial diagnosis and disease recurrence, prior treatment, and lymphoma and symptom burden at relapse. 

Future strategies in the first-line treatment of NLPHL should aim at reducing treatment intensity whenever possible while maintaining the efficacy achieved with current approaches. To this end, novel tools allowing a more precise definition of risk groups are required. Based on a refined risk group allocation that may implement clinical presentation, pathological characteristics, and imaging results, low-risk patients will ideally be treated sufficiently with few cycles of non-intensive chemotherapy or chemoimmunotherapy optionally followed by RT, whereas a small group of high-risk patients will likely receive more intensive chemotherapy supplemented by rituximab. In relapsed NLPHL, a more concise identification of the few patients who benefit from intensive salvage treatment with high-dose chemotherapy and ASCT is needed to prevent overtreatment in patients who do not necessitate intensive salvage therapy. International collaborations between study groups and other institutions are required to achieve these goals.

## Figures and Tables

**Table 1:** Treatment options in newly diagnosed NLPHL.

Stage	Treatment	PFS	OS	Reference
	**RT alone**			
Stage IA	RT alone	8-year PFS: 84.3% (EF-RT) 91.9% (IF-RT)	8-year OS: 95.7% (EF-RT) 99% (IF-RT)	[16]
Stage I	RT alone	10-year PFS: 89%	10-year OS: 96%	[18]
Stage II	RT alone	10-year PFS: 72%	10-year OS: 100%	[18]
Stages I/II	RT alone	5-year PFS: 91.1%	5-year PFS: 99.4%	[24]
Limited-stage	RT alone	10-year PFS: 65%	10-year OS: 84%	[25]
	**HL-directed approaches**			
Limited-stage	ABVD(-like) chemotherapy plus RT	10-year PFS: 91%	10-year OS: 93%	[25]
Stage I/II	Combined-modality treatment	5-year PFS: 90.5%	5-year PFS: 99.4%	[24]
Early stage favorable	ABVD(-like) chemotherapy plus RT	10-year PFS: 79.7%	10-year OS: 93.3%	[26]
Early stage unfavorable	ABVD(-like) or BEACOPP variants plus RT	10-year PFS: 72.1%	10-year OS: 96.2%	[26]
Stages II–IV	ABVD ± RT	5-year PFS: 72.7%	5-year OS: 95.0%	[27]
Advanced	ABVD(-like) ± RT	10-year TTP: 63%	10-year OS: 83.5%	[28]
Advanced	BEACOPP variants ± RT	10-year PFS: 69.8%	10-year OS: 87.4%	[26]
	**Rituximab-containing and NHL-directed approaches**			
Stage IA	Rituximab alone	10-year PFS: 51.1%	10-year OS: 91%	[29]
All stages	Rituximab alone	5-year PFS: 41.7%	5-year OS: 100%	[30]
All stages	Rituximab induction plus rituximab maintenance	5-year PFS: 51.9%	5-year OS: 100%	[30]
Stages II–IV	Rituximab plus chemotherapy (ABVD or CHOP)	5-year PFS: 89.6%	5-year OS: 98.8%	[27]
All stages	Rituximab plus bendamustine	After 74 months median FU (20 pts): no relapse	After 74 months median FU (20 pts): no death	[31]

Legend: PFS: progression-free survival; OS: overall survival; RT: radiotherapy; EF: extended-field; IF: involved-field; HL: Hodgkin lymphoma; TTP: time to progression; NHL: non-Hodgkin lymphoma; FU: follow-up; pts: patients.

**Table 2:** Treatment options in relapsed NLPHL.

Treatment	PFS	OS	Reference
**Anti-CD20 antibody treatment alone or RT alone**			
Rituximab alone	5-year PFS: 36.4%	5-year OS: 90.9%	[30]
Rituximab induction plus rituximab maintenance	5-year PFS: 71.4%	5-year OS: 71.4%	[30]
Ofatumumab alone	2-year PFS: 80%	2-year OS: 100%	[41]
Rituximab alone (or RT alone)	5-year PFS: 74.1%	5-year OS: 97.2%	[43]
RT alone	5-year PFS: 70%	5-year OS: n/a	[44]
**Conventional chemotherapy**			
Conventional chemotherapy ± rituximab ± RT	5-year PFS: 68%	5-year OS: 77.8%	[43]
Conventional chemotherapy ± RT	5-year PFS: 58%	5-year OS: n/a	[44]
**High-dose chemotherapy and ASCT**			
High-dose chemotherapy and ASCT	5-year PFS: 84.6%	5-year OS: 89.8%	[43]
High-dose chemotherapy and ASCT	5-year PFS: 83%	5-year OS: n/a	[44]
High-dose chemotherapy and ASCT	5-year OS: 66%	5-year OS: 87%	[45]
High-dose chemotherapy and ASCT	5-year EFS: 69%	5-year OS: 76%	[46]

Legend: PFS: progression-free survival; OS: overall survival; RT: radiotherapy; ASCT: autologous stem cell transplantation; EFS: event-free survival; n/a: not available.
